# Supplementary material for: The Efficacy of Faecal Microbiota Transplant and Rectal Bacteriotherapy in Patients with Recurrent Clostridioides difficile Infection: A Retrospective Cohort Study
Source: Cells. 2022 Oct 18;11(20):3272. doi: 10.3390/cells11203272 (PMC9600246; doi:10.3390/cells11203272)
Supplement: Supplementary file 1 [file cells-11-03272-s001.zip › cells-1910758-supplementary.pdf]

cells MDPI

Supplementary Materials

1. Definitions

CDI: three or more loose stools daily (Bristol stool chart 6-7) and a positive polymerase chain reaction (PCR) test [1].

Clinical resolution from Clostridioides difficile infection (CDI) (in this cohort study): absence of diarrhoea or a negative polymerase chain reaction test (PCR test) for *Clostridioides difficile* eight weeks after intestinal microbiota transplantation.

Charlson Comorbidity Index (CCI): a method for classifying comorbid conditions and to estimate risk of death from disease [2, 3].

Diarrhoea: three or more loose or liquid bowel movements a day (Bristol Stool Chart 6-7).

Fulminant CDI: fever ≥ 38.5°C, cognitive impairment, hypotension, ileus, megacolon, pseudomembranous colitis or signs of organ failure [1].

Immunosuppressive disease: any disease or illness in, or dysfunction of, the innate or adaptive immune system.

Immunosuppressive medication: any medication that is known to cause increased risk of infection by inhibiting the immune response, e.g chemotherapy, immunomodulatory medication including prednisone (despite inhalation and topical cremes).

Intestinal microbiota transplantation: either Fecal Microbiota Transplant (FMT) or Rectal bacteriotherapy (RBT)

Mild/moderate CDI: clinical symptomatic diarrhoa and none of the following: albumin ≤ 30 g/L, Leukocytes < 2 x10^9/L or >15 x10^9/L, abdominal, renal failure, fever ≥ 38.5°C, cognitive impairment, hypotension, ileus, megacolon, pseudomembranous colitis or signs of organ failure [1].

Previous CDI episode: any CDI prior to the current event leading up to FMT or RBT treatment. In Table S1, number of CDI infections is divided into subgroups 'first CDI and 1st recurrence', '2nd recurrence' and '≥3rd recurrence'.

Recurrent Clostridioides difficile infection (rCDI) (in this cohort study): recurrence of diarrhoea following initial treatment response and a PCR test after cessation of treatment at any timepoint after initial clinical resolution.

Refractory CDI: *either* clinical deterioration of diarrhoa symptoms and development of signs of severe/fulminant CDI during the first six days of treatment *or* absence of clinical response despite ongoing treatment after six days of treatment [1].

Severe CDI: albumin ≤ 30 g/L, Leukocytes < 2 x10^9/L or >15 x10^9/L, abdominal pain or renal failure [1].

2. Supplementary figures and tables

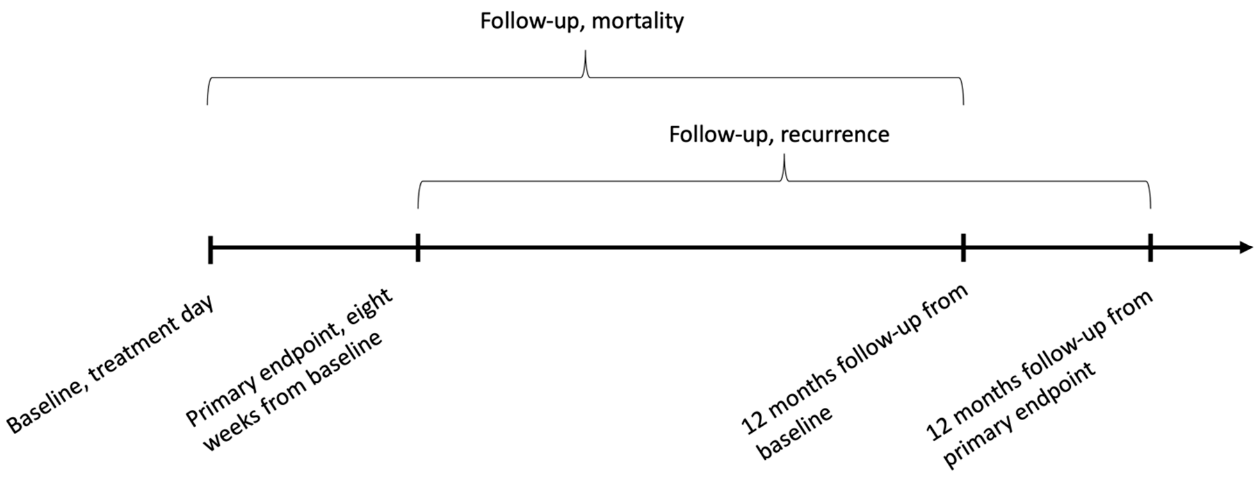

Figure S1. Follow-up. Follow-up for new recurrence in patients who initially had clinical resolution eight weeks after treatment, and follow-up for mortality including patients who died before meeting the primary endpoint.

Table S1. Clinical resolution from CDI in the three treatment groups subdivided according to the number of previous CDI episodes

No. of CDI recurrences	FMT capsules N=69 (%)	FMT enema N=92 (%)	RBT N=173 (%)	OR (95% CI)	p	Adjust. OR [d] (95% CI)	p
Any	55 of 69	49 of 92	107 of 173	3.45 (1.71 to 7.24) [a]	<0.001 [a]	3.79 (1.82 to 8.26) [a]	<0.001 [a]
	(79.7)	(53.3)	(61.8)	2.42 (1.28 to 4.84) [b]	<0.01 [b]	2.92 (1.49 to 6.03) [b]	<0.01 [b]
				1.09 (0.51 to 2.45) [c]	0.28 [c]	1.29 (0.74 to 2.27) [c]	0.36 [c]
Initial CDI or 1st recurrence	10 of 11	4 of 12	8 of 17	20.00 (2.53 to 440.91) [a]	0.014 [a]	33.98 (3.45 to 926.70) [a]	0.009 [a]
	(90.1)	(33.3)	(47.05)	11.25 (1.61 to 230.81) [b]	0.04 [b]	26.01 (2.88 to 654.16) [b]	0.012 [b]
				1.78 (0.39 to 8.86) [c]	0.46 [c]	1.31 (0.22 to 8.16) [c]	0.77 [c]
2nd recurrence	27 of 31	20 of 31	46 of 66	3.71 (1.09 to 15.00) [a]	0.045 [a]	4.33 (1.17 to 19.12) [a]	0.04 [a]
	(87.1)	(64.5)	(69.7)	2.93 (0.98 to 10.89) [b]	0.07 [b]	3.27 (1.00 to 13.07) [b]	0.06 [b]
				1.27 (0.50 to 3.11) [c]	0.61 [c]	1.32 (0.48 to 3.55) [c]	0.58 [c]
≥3RD recurrences	18 of 27	25 of 49	53 of 90	1.92 (0.73 to 5.26) [a]	0.19 [a]	1.85 (0.69 to 5.19) [a]	0.23 [a]
	(66.7)	(51.0)	(58.9)	1.40 (0.58 to 3.57) [b]	0.47 [b]	1.50 (0.61 to 3.91) [b]	0.39 [b]
				1.38 (0.68 to 2.78) [c]	0.37 [c]	1.23 (0.60 to 2.56) [c]	0.57 [c]

Adjust. OR, adjusted odds ratio. [a] FMT capsules compared to enema; [b] FMT capsules compared to RBT; [c] RBT compared to FMT enema, [d] The odds ratio in the "any" group is adjusted for age, gender, CCI, number of previous CDI's, *Clostridioides difficile* subtype, severity of current CDI and the duration of antibiotic treatment leading up to intestinal microbiota treatment. Odds ratios in the subgroups 'Initial CDI or 1st recurrence', '2nd recurrence' and '⩾3rd recurrence' are only adjusted for age, gender, CCI, and the duration of antibiotic treatment leading up to intestinal microbiota treatment, because of small numbers.

Table S2. Clinical resolution subdivided into the year of treatment

	FMT capsules No. (%)	FMT enema No. (%)	RBT No. (%)	OR (95% CI)	p	Adjust. OR [d] (95% CI)	p
2017		12 of 17 (70.6)	16 of 25 (64.0)	0.74 (0.89 to 7.54) [c]	0.66 [c]	0.60 (0.12 to 2.65) [c]	0.51 [c]
2018		14 of 26 (53.8)	12 of 21 (57.1)	1.14 (0.36 to 3.70) [c]	0.82 [c]	-	
2019		10 of 22 (45.5)	25 of 43 (58.1)	1.17 (0.59 to 4.78) [c]	0.33 [c]	1.51 (0.45 to 5.23) [c]	0.50 [c]
2020	4 of 7 (57.1)	9 of 16 (56.2)	27 of 42 (66.7)	1.04 (0.17 to 6.77) [a]	0.97 [a]		
				0.67 (0.13 to 3.77) [b]	0.63 [b]	-	
				1.56 (0.47 to 5.08) [c]	0.46 [c]		
2021	51 of 62 (82.3)	4 of 11 (36.4)	26 of 42 (61.9)	8.11 (2.10 to 35.83) [a]	0.003 [a]	11.97 (2.49 to 69.80) [a]	0.003 [a]
				2.85 (1.17 to 7.19) [b]	0.02 [b]	3.39 (1.28 to 9.42) [b]	0.015 [b]
				2.84 (0.74 to 12.34) [c]	0.14 [c]	3.53 (0.75 to 19.09) [c]	0.12 [c]

Adjust. OR, adjusted odds ratio. [a] FMT capsules compared to enema; [b] FMT capsules compared to RBT; [c] RBT compared to FMT enema, [d] The odds ratio is adjusted for gender, age, CCI, *Clostridioides difficile* subtype, severity of illness, duration of antibiotic treatment and previous CDI episodes. It was not possible to calculate the adjusted OR for year 2018 and 2020 because of small numbers.

Table S3. Clinical resolution subdivided into place of manufacturing

Treatment modalities	OR (95% CI)	p	Adjust. OR [d] (95% CI)	p
Without FMT produced at CEFTA	3.15 (1.35 to 7.92) [a]	0.01 [a]	3.60 (CI 1.45 to 9.60) [a]	0.007 [a]
	2.40 (1.12 to 5.60) [b]	0.03 [b]	3.11 (CI 1.39 to 7.60) [b]	0.008 [b]
	1.31 (0.74 to 2.33) [c]	0.35 [c]	1.16 (CI 0.61 to 2.18) [c]	0.64 [c]
Without FMT enema produced at CEFTA	3.19 (1.51 to 6.96) [a]	0.003 [a]	3.46 (1.56 to 7.96) [a]	0.003 [a]
	2.42 (1.28 to 4.84) [b]	0.008 [b]	3.11 (1.39 to 7.60) [b]	0.008 [b]
	1.31 (0.74 to 2.32) [c]	0.35 [c]	1.22 (0.65 to 2.30) [c]	0.52 [c]
Without FMT capsules produced at CEFTA	3.41 (1.52 to 8.28) [a]	0.004 [a]	3.95 (1.69 to 9.97) [a]	0.002 [a]
	2.40 (1.12 to 5.60) [b]	0.03 [b]	3.09 (1.40 t0 7.44) [b]	0.008 [b]
	1.42 (0.85 to 2.38) [c]	0.18 [c]	1.28 (0.73 to 2.24) [c]	0.39 [c]
Without FMT produced at CUHH	4.33 (1.29 to 16.45) [a]	0.02 [a]		
	2.47 (0.95 to 7.70) [b]	0.08 [b]	-	
	1.76 (0.75 to 4.13) [c]	0.19 [c]		

Adjust. Adjust. OR, adjusted odds ratio; CEFTA, Centre for Fecal Transplantation; CUHH, Copenhagen University Hospital Hvidovre; FMT, Fecal Microbiota Transplantation. [a] FMT capsules versus FMT enema, [b] FMT capsules versus RBT, [c] RBT versus FMT enema, [d] Odds ratio is adjusted for gender, age, CCI, *Clostridioides difficile* subtype, severity of illness, duration of antibiotic treatment and previous CDI episodes. It was not possible to calculate the adjusted OR in the subgroup without FMT produced at CUHH because of small numbers.

Table S4. Cumulative prevalence of recurrence after initial clinical resolution, subdivided into time periods

Time of recurrence	FMT capsules N=55 (%)	FMT enema N=49 (%)	RBT N=107 (%)	HZ (95% CI)	p	Adjust. HZ (95% CI) [d]	p
Any time	3 of 55 (5.5)	13 of 49 (26.5)	28 of 107 (26.2)	0.32 (0.09 to 1.16) [a]	0.08 [a]	0.31 (0.08 to 1.13) [a]	0.08 [a]
				0.30 (0.09 to 1.00) [b]	0.049 [b]	0.29 (0.08 to 0.98) [b]	0.046 [b]
				1.08 (0.56 to 2.10) [c]	0.81 [c]	1.07 (0.55 to 2.10) [c]	0.84 [c]
0-6 months	3 of 55 (5.5)	5 of 49 (10.2)	16 of 107 (15.0)	0.54 (0.13 to 2.27) [a]	0.40 [a]	0.46 (0.10 to 2.00) [a]	0.30 [a]
				0.36 (0.11 to 1.24) [b]	0.11 [b]	0.32 (0.09 to 1.15) [b]	0.08 [b]
				1.50 (0.55 to 4.09) [c]	0.43 [c]	1.42 (0.52 to 3.92) [c]	0.49 [c]
0-12 months	3 of 55 (5.5)	11 of 49 (22.4)	22 of 107 (20.6)	0.30 (0.08 to 1.09) [a]	0.07 [a]	0.24 (0.06 to 0.89) [a]	0.03 [a]
				0.32 (0.09 to 1.06) [b]	0.06 [b]	0.26 (0.08 to 0.91) [b]	0.04 [b]
				0.96 (0.46 to 1.97) [c]	0.90 [c]	0.89 (0.43 to 1.87) [c]	0.77 [c]

Adjust. HZ, adjusted hazard ratio. [a] FMT capsules compared to enema; [b] FMT capsules compared to RBT; [c] RBT compared to FMT enema; [d] hazard ratio is adjusted for age, gender, CCI, number of previous CDI's, *Clostridioides difficile* subtype, and the duration of antibiotic treatment leading up to intestinal microbiota treatment

References

1. Baunwall, S.M.D.; Dahlerup, J.F.; Engberg, J.H.; Erikstrup, C.; Helms, M.; Juel, M.A.; Kjeldsen, J.; Nielsen, H.L.; Nilsson, A.C.; Rode, A.A.; Vinter-Jensen, L.; Hvas, C.L. Danish national guideline for the treatment of Clostridioides difficile infection and use of faecal microbiota transplantation (FMT). *Scand J Gastroenterol* **2021**, *56*, p. 1056-1077.
2. Charlson, M.; Szatrowski, T.P.; Peterson, J.; Gold, J. Validation of a combined comorbidity index. *J Clin Epidemiol* **1994**, *47*, p. 1245-51.
3. Charlson, M.E.; Pompei, P.; Ales, K.L.; MacKenzie, C.R. A new method of classifying prognostic comorbidity in longitudinal studies: development and validation. *J Chronic Dis* **1987**, *40*, p. 373-83.
